# Sugar as an Anthelmintic

**Published:** 1863-11

**Authors:** 


Sugar as an Anthelmintic.—M. Debout says that sugar is an excellent destroyer of worms. He once accidentally put sugar instead of salt on a leech which he wished to detach from the skin, and was surprised at the spasms produced by it. He therefore tried sugar on earth-worms, and found it had a similar powerful effect; and has since used it in solution with success as an injection in children.—*British Med. Journal.*

